# Laser Speckle Imaging of Rat Pial Microvasculature during Hypoperfusion-Reperfusion Damage

**DOI:** 10.3389/fncel.2017.00298

**Published:** 2017-09-25

**Authors:** Teresa Mastantuono, Noemy Starita, Laura Battiloro, Martina Di Maro, Martina Chiurazzi, Gilda Nasti, Espedita Muscariello, Mario Cesarelli, Luigi Iuppariello, Gianni D’Addio, Alexander Gorbach, Antonio Colantuoni, Dominga Lapi

**Affiliations:** ^1^Department of Clinical Medicine and Surgery, “Federico II” University Medical School Naples, Italy; ^2^Molecular Biology and Viral Oncology Unit, Istituto Nazionale Tumori IRCCS—“Fondazione G.Pascale” Naples, Italy; ^3^Department of Biomedical, Electronics and TLC Engineering, University of Naples, “Federico II” Naples, Italy; ^4^IRCCS S. Maugeri Foundation Benevento, Italy; ^5^Infrared Imaging & Thermometry Unit, NIBIB, National Institutes of Health Bethesda, MD, United States

**Keywords:** laser speckle imaging, bilateral common carotid artery occlusion, reperfusion, pial microcirculation, blood flow oscillations, spectral analysis, frequency components, myogenic activity

## Abstract

The present study was aimed to *in vivo* assess the blood flow oscillatory patterns in rat pial microvessels during 30 min bilateral common carotid artery occlusion (BCCAO) and 60 min reperfusion by laser speckle imaging (LSI). Pial microcirculation was visualized by fluorescence microscopy. The blood flow oscillations of single microvessels were recorded by LSI; spectral analysis was performed by Wavelet transform. Under baseline conditions, arterioles and venules were characterized by blood flow oscillations in the frequency ranges 0.005–0.0095 Hz, 0.0095–0.021 Hz, 0.021–0.052 Hz, 0.052–0.150 Hz and 0.150–0.500 Hz. Arterioles showed oscillations with the highest spectral density when compared with venules. Moreover, the frequency components in the ranges 0.052–0.150 Hz and 0.150–0.500 were predominant in the arteriolar total power spectrum; while, the frequency component in the range 0.150–0.500 Hz showed the highest spectral density in venules. After 30 min BCCAO, the arteriolar spectral density decreased compared to baseline; moreover, the arteriolar frequency component in the range 0.052–0.150 Hz significantly decreased in percent spectral density, while the frequency component in the range 0.150–0.500 Hz significantly increased in percent spectral density. However, an increase in arteriolar spectral density was detected at 60 min reperfusion compared to BCCAO values; consequently, an increase in percent spectral density of the frequency component in the range 0.052–0.150 Hz was observed, while the percent spectral density of the frequency component in the range 0.150–0.500 Hz significantly decreased. The remaining frequency components did not significantly change during hypoperfusion and reperfusion. The changes in blood flow during hypoperfusion/reperfusion caused tissue damage in the cortex and striatum of all animals. In conclusion, our data demonstrate that the frequency component in the range 0.052–0.150 Hz, related to myogenic activity, was significantly impaired by hypoperfusion and reperfusion, affecting cerebral blood flow distribution and causing tissue damage.

## Introduction

Laser speckle imaging (LSI) is a real-time, dynamic and non invasive technique, that permits the high-resolution mapping of tissue blood perfusion in a non-contact and thus non-perturbing manner.

Laser speckle is a random interference pattern, produced by the coherent addition of scattered laser light. Particularly, when an optically rough object is illuminated by a coherent laser, the light is reflected or scattered from different parts of the illuminated surface with varying path lengths, producing a speckle pattern. Moreover, if the scattering object is moving, the speckle pattern fluctuates in intensity. These time-varying speckle fluctuations can be analyzed by temporal and spatial statistics, providing information about the movement of the scattering object (Briers, 2001). Consequently, two-dimensional maps of tissue blood perfusion can be obtained with very high spatial and temporal resolution.

LSI technique, introduced by Fercher and Briers, has been widely utilized to evaluate blood flow in different tissues, such as retina, skin and brain (Boas and Dunn, 2010; Dunn, 2012; Mahé et al., 2012). Additionally, several studies have demonstrated that LSI is a valid method useful in the *in vivo* study of rat cerebral blood flow under both physiological and pathological conditions (Durduran et al., 2004; Kharlamov et al., 2004; Zhu et al., 2007; Li et al., 2009). This technique, indeed, offers an advantage over conventional methods, including laser Doppler perfusion monitoring (LDPM) and laser Doppler imaging, providing information about cerebral blood flow perfusion from a limited number of isolated points in the brain (1 mm^3^).

Up to now in several studies LSI has been used to monitor the temporal and spatial changes in blood flow oscillatory patterns of vascular beds. Scully et al. (2013, 2014) have demonstrated that LSI measurements can be used to study changes in renal blood flow, due to local and global dynamics generated by various physiological stimuli (Mitrou et al., 2015). However, there are no data about cerebral blood flow in single vessels under baseline conditions and after brain hypoperfusion and reperfusion.

Rhythmic oscillations in blood flow have been observed in several experimental models or clinical settings by LDPM. In rat brain, it has been reported that oscillatory patterns characterize pial blood flow (Hudetz et al., 1992; Ursino et al., 1998; Mastantuono et al., 2016a). However, a large number of studies have shown that these oscillations in blood flow are due to the several physiological factors, regulating microvascular blood flow redistribution (Kvandal et al., 2003, 2006; Lapi et al., 2010).

Our previous results indicate that rat pial circulation presents oscillation in arteriolar diameter, due to the same mechanisms reported in human cutaneous microcirculation. In particular, six frequency components have been identified in the ranges 0.001–4.5 Hz. Four low frequency components in the ranges 0.001–0.0095 Hz, 0.0095–0.02 Hz, 0.02–0.06 Hz and 0.06–0.2 Hz were related to NO-independent and NO-dependent endothelial activity, to the neurogenic and to the intrinsic myogenic activity of vascular smooth muscle cells, respectively. Two high frequency components into ranges 0.2–2.0 Hz and 2.0–4.5 Hz have been associated to the respiration and the heart rates, respectively (Lapi et al., 2017). Therefore, the aim of the present study was to *in vivo* investigate the rat pial microcirculation by LSI; in particular, we tried to evaluate the frequency components in blood flow oscillations during brain hypoperfusion and reperfusion.

At first, LSI results were compared with data obtained by fluorescent microscopy technique to validate the use of LSI for assessing pial microcirculation. Therefore, LSI and fluorescent microscopy were carried out on rat pial microvasculature, recording the same cerebral area to obtain comparable data and to evaluate the LSI resolution. For this purpose, we classified arterioles and venules according to the Strahler’s method, to characterize the microvascular networks under baseline conditions (Kassab et al., 1993, 1994; Lapi et al., 2008). Moreover, the microvascular blood flow changes, due to 30 min hypoperfusion and 60 min reperfusion, were monitored by the two methods. Microvascular damage was assessed by the changes in arteriolar diameter, microvascular permeability, leukocyte adhesion, single pial venule (SPV) blood flow and perfused capillary length (PCL) using fluorescent microscopy. In addition, we estimated the neuronal damage by 2,3,5-triphenyltetrazolium chloride (TTC) staining.

In the same experimental model, we assessed the changes in the overall blood perfusion by LSI. However, the main purpose of the present study was to evaluate the blood flow oscillatory patterns and the related frequency components in single vessels during the different experimental conditions: under baseline conditions, after 30 min bilateral common carotid artery occlusion (BCCAO) and at 60 min reperfusion. The blood flow oscillations, recorded in single arterioles and venules under baseline conditions and during hypoperfusion-reperfusion, were analyzed by spectral methods, such as Wavelet transform.

## Materials and Methods

### Experimental Groups

Male Wistar rats (250–300 g) were randomly assigned to the following groups: (1) sham-operated group (S group, *n* = 12), subjected to the same surgical procedures as the other experimental group without hypoperfusion-reperfusion; (2) the second group (H group, *n* = 12), submitted to 30 min BCCAO plus 60 min reperfusion.

In each group nine rats were used for microvascular studies, while three animals were utilized to evaluate neuronal damage by TTC staining.

### Animal Preparation

All experiments conform to the *Guide for the Care and Use of Laboratory Animals* published by the US National Institutes of Health (NIH Publication No. 85-23, revised 1996) and to institutional rules for the care and handling of experimental animals. The protocol was approved by the “Federico II” University of Naples Ethical Committee.

After an overnight food restriction, anesthesia was induced with α-chloralose (50 mg/Kg b.w., i.p.) and maintained with supplemental injections of α-chloralose (30 mg/Kg b.w., i.v., every hour). Animals were paralyzed with tubocurarine chloride (1 mg/Kg·h, i.v.), tracheotomized and mechanically ventilated with room air and supplemental oxygen. The right and left common carotid arteries were isolated for successive clamping. Then, the left femoral artery and the right femoral vein were catheterized: the arterial catheter was used to measure arterial blood pressure and blood gases. The venous catheter was utilized to administer additional anesthesia and fluorescent tracers [fluorescein isothiocyanate bound to dextran, molecular weight 70 kDa (FD 70), 50 mg/100 g b.w., i.v. as 5% wt/vol solution in 3 min administered just once at the beginning of experiment after 30 min of the preparation stabilization; rhodamine 6G, 1 mg/100 g b.w. in 0.3 ml, as a bolus with supplemental injection throughout BCCAO and reperfusion (final volume 0.3 ml·100 g^−1^·h^−1^)]. Blood gas measurements were carried out on arterial blood samples withdrawn from arterial catheter at 30 min time period intervals. Throughout all experiments, rats were secured on a heating stereotaxic frame to preserve the animal temperature at 37.0 ± 0.5°C. Core body temperature was monitored through a rectal probe. Moreover, mean arterial blood pressure (MABP), heart rate, respiratory CO_2_ and blood gases values were recorded and maintained stable within physiological ranges.

To observe the pial microcirculation, a cranial window (4 × 5 mm) was prepared above the left frontoparietal cortex (posterior 1.5 mm to bregma; lateral, 3 mm to the midline), according to the method previously described (Ngai et al., 1988). Briefly, a 1 cm incision was made in the skin to expose the skull and a craniotomy was performed. The dura mater was gently removed and a 150-μm-thick quartz microscope coverglass was sealed to the bone with dental cement. The window inflow and outflow were assured by two needles secured in the walls of the skin, adjusted to a well, so that the pia mater was continuously superfused with artificial cerebrospinal fluid (aCSF; Hudetz et al., 1985; Morii et al., 1986). The rate of superfusion was 0.5 ml/min controlled by a peristaltic pump. The composition of the aCSF was: 119.0 mM NaCl, 2.5 mM KCl, 1.3 mM MgSO_4_·7 H_2_O, 1.0 mM NaH_2_PO_4_, 26.2 mM NaHCO_3_, 2.5 mM CaCl_2_ and 11.0 mM glucose (equilibrated with 10.0% O_2_, 6.0% CO_2_ and 84.0% N_2_; pH 7.38 ± 0.02). The temperature was maintained at 37.0 ± 0.5°C with a water bath.

Hypoperfusion was obtained by placement of two atraumatic microvascular clips on common carotid arteries, previously isolated. After 30 min, the clamps were removed and the pial microcirculation was observed for 60 min (reperfusion). During this period microvascular responses were studied.

### Fluorescence Intravital Microscopy

Pial microcirculation was observed by a fluorescence microscope (LeitzOrthoplan) fitted with long-distance objectives [2.5×, numerical aperture (NA) 0.08; 10×, NA 0.20; 20×, NA 0.25; 32×, NA 0.40], a 10× eyepiece and a filter block (Ploemopak, Leitz). Epi-illumination was provided by a 100 W mercury lamp using the appropriate filters for FITC, for rhodamine 6G and a heat filter (LeitzKG1). The pial microcirculation was televised with a DAGE MTI 1000 low-light level camera and recorded by a computer based frame grabber (Pinnacle DC 10 plus, Avid Technology, MA, USA).

**Figure 1:**
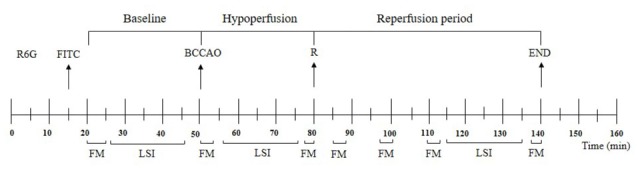
Experimental design and recording times. R6G, rhodamine 6G; FITC, fluorescein isothiocyanate bound to dextran (70 kDa); BCCAO, bilateral common carotid artery occlusion; R, beginning of reperfusion; FM, recording of microvascular images by fluorescent microscopy; LSI, recording by laser speckle imaging.

Microvascular measurements were made off-line using a computer-assisted imaging software system (MIP Image, CNR, Institute of Clinical Physiology, Pisa, Italy). Recording of microvascular images was carried out for 5 min during baseline, for 6 min during BCCAO (1–3 min, 27–30 min) and 12 min during reperfusion (5–8 min, 18–21 min, 30–33 min, 58–60 min), according to the protocol time schedule reported in Figure 1. The baseline conditions were represented by microvascular values detected within 2 min of FITC administration.

### Microvascular Parameter Evaluation

Under baseline conditions, the microvascular networks were mapped by stop-frame images, so that pial arterioles were classified according to a centripetal ordering scheme (Strahler’s method, modified according to diameter). Order 0 was assigned to the capillaries; thereafter, the terminal arterioles were assigned order 1 and the vessels upstream were assigned progressively higher order, as previously described. Conversely, the pial venules were classified from the smaller order to the larger ones, assigning −1 order to the smallest and −5 to the largest vessels in the preparation (Kassab et al., 1993, 1994; Lapi et al., 2008).

Vessel diameters were measured with a computer-assisted method (MIP Image program, frame by frame). The results of diameter measurements were in accord with those obtained by shearing method (±0.5 μm). To avoid bias due to single operator measurements, two independent “blinded” operators measured the vessel diameters. Their measurements overlapped in all cases.

The increase in permeability was calculated and reported as normalized gray levels (NGL): NGL = (I − Ir)/Ir, where Ir is the average baseline gray level at the end of vessel filling with fluorescence (average of five windows located outside the blood vessels with the same windows being used throughout the experimental procedure) and I is the same parameter at the end of BCCAO or at the end of reperfusion (RE). Gray levels ranging from 0 to 255 were determined by the MIP Image program in five regions of interest (ROI) measuring 50 × 50 μm (10× objective). The same location of ROI during recordings along the microvascular networks was provided by a computer-assisted device for XY movement of the microscope table.

Adherent leukocytes (i.e., cells on vessel walls that did not move over a 30-s observation period) were quantified in terms of number/100 μm of venular length (v.l.)/30 s using higher magnification (20× and 32×, microscope objectives). In each experimental group 25 venules were studied. Moreover, for each experimental group 19 pial venules were studied to evaluate the SPV blood flow, Q, calculated according to the following equation: *Q* = α × *V*_CL_ × *A*, where α was a constant related to the vessel diameter, *V*_CL_ was the red blood cell centerline velocity and *A* was the cross-section area.

PCL was measured by MIP image in an area of 150 × 150 μm. In this system the length of perfused capillaries is easily established by the automated process because it is outlined by dextran (Colantuoni et al., 2005).

MABP (Viggo-Spectramed P10E2 trasducer, Oxnard, CA, USA, connected to a catheter in the femoral artery) and heart rate were monitored with a Gould Windograf recorder (model 13-6615-10S, Gould, OH, USA). Data were recorded and stored in a computer. Blood gas measurements were carried out on arterial blood samples withdrawn from arterial catheter (ABL5; Radiometer, Copenhagen, Denmark). The hematocrit was measured under baseline conditions, at the end of BCCAO and at RE.

### Laser Speckle Imaging

LSI measurements were carried out using the MOOR-FLPI laser speckle contrast imager (Moor Instruments, Axminster, UK) with an increased optical zoom and exposure time set to 2 ms. A 775-nm laser was used to illuminate the pial microcirculation and laser speckle images were acquired using a 568 × 760-pixel grayscale charge-coupled device camera at a sampling rate of 1 Hz. Using 5 × 5 pixel window to calculate speckle contrast, the maximal image resolution was 5 μm/pixel. MOOR-FLPI translates the speckle contrast value into a relative perfusion unit giving information about blood flow, related to velocity and/or concentration of red blood cells. Pseudo-color images with the levels of perfusion scaled from blue (low perfusion) to red (high perfusion) were obtained. LSI recording was performed for 20 min during baseline subsequent to FITC administration, during hypoperfusion (6–26 min) and after 40 min reperfusion (35–55 min).

### Laser Speckle Imaging Analysis

The recorded images were stored in a multiband file with a band-sequential arrangement. In a multiband file the images were stored in a 3-D array. In our file a sequence of the same anatomical structures recorded at subsequent times was stored (the *z*-axis corresponds to the time axis). A Matlab routine was developed to extract the 3D array (using the Matlab “multibandread” function), to select points on the image corresponding to the arterioles or venules and to store their locations (corresponding to the X and Y coordinates on the 3D image array). The perfusion signals were extracted from the array, build a vector containing all values at the same X, Y coordinates (corresponding to the selected anatomical site). Then, a preprocessing was performed which included a linear detraining of the signal, thus eliminating the average component, a moving average filter with a time window of about 200 s, to remove trends below 0.0025 Hz, and a second moving average filtering with a time window of about 0.25 s to remove the trends above 2 Hz. Further, the dynamics of blood flow in the microcirculation were processed using Wavelet (Morlet mother wave was used with a center frequency of 0.8125 Hz), perfusion signals obtaining the total spectrum of the signal. Finally, the spectrum was integrated within five frequency bands (0.005–0.0095 Hz, 0.0095–0.021 Hz, 0.021–0.052 Hz, 0.052–0.150 Hz and 0.150–0.500 Hz) and the power of each frequency band as a parameter, expressed as percent of the total power.

### TTC Staining

Rats were sacrificed after 30 min BCCAO and 60 min reperfusion. Tissue damage was evaluated by TTC staining. The brains were cut into 1 mm coronal slices with a vibratome (Campden Instrument, 752 M; Lafayette, IN, USA). Sections were incubated in 2% TTC for 20 min at 37°C and in 10% formalin overnight. The necrotic area site and extent in each section were evaluated by image analysis software (Image-Pro Plus; Rockville, MD, USA; Bederson et al., 1986).

### Statistical Analysis

All data were expressed as mean ± SEM. Data were tested for normal distribution with the Kolmogorov-Smirnov test. Parametric (Student’s *t* tests, ANOVA and Bonferroni *post hoc* test) or nonparametric tests (Wilcoxon, Mann-Whitney and Kruskal-Wallis tests) were used; nonparametric tests were applied to compare diameter and length data among experimental groups. The statistical analysis was carried out by SPSS 14.0 statistical package. Statistical significance was set at *p* < 0.05.

## Results

### Baseline Observations

**Figure 2:**
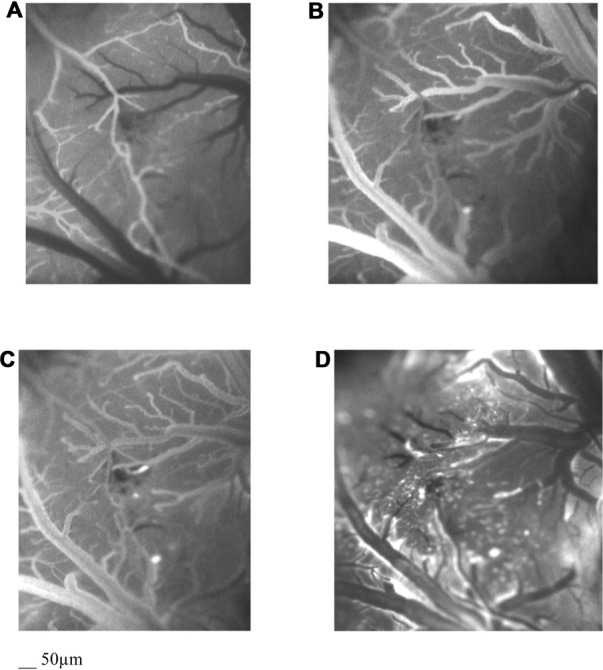
Computer-assisted images by fluorescent microscopy of the arteriolar **(A)** and the venular **(B)** pial network under baseline conditions, at the end of BCCAO **(C)** and at the end of reperfusion **(D)** in a hypoperfused rat. The increase in microvascular leakage is outlined by the marked change in the color of interstitium (from black to white).

Under baseline conditions, the morphological characteristics of the pial microvascular networks were defined using fluorescence microscopy (Figures 2A,B). In particular, in all experimental preparations, arterioles were classified from the largest vessels, assigned order 5 (average diameter 62.1 ± 5.2 μm), to the smallest ones supplying blood to the capillaries, assigned order 1 (average diameter: 16.1 ± 2.5 μm). Capillaries, sprouting from order 1 arterioles, were assigned order 0, as previously reported (Table 1). Finally, venules were classified from the smallest vessels draining the capillaries, assigned order −1 (average diameter 23 ± 3 μm), to the largest ones, assigned order −5 (average diameter 90 ± 7 μm; Table 2). Therefore, detailed maps of each rat pial microcirculation were obtained.

**Table 1:** Diameter and length of each arteriolar order in all animals.

Order	Arteriole (*n*)	Diameter (μm)	Length (μm)	Rats (*n*)
5	20	62.1 ± 5.2*	1250 ± 209	22
4	35	44.8 ± 3.8*	990 ± 185	22
3	46	35.2 ± 4.0*	460 ± 95	22
2	62	24.6 ± 2.8*	368 ± 55	22
1	94	16.1 ± 2.5*	150 ± 39	22

*p < 0.01 vs. different order.

**Table 2:** Diameter and length of each venular order in all animals.

Order	Venule (*n*)	Diameter (μm)	Length (μm)	Rats (*n*)
−1	48	23 ± 3*	80 ± 9	22
−2	72	36 ± 5*	220 ± 15	22
−3	85	44 ± 4*	560 ± 18	22
−4	38	68 ± 6*	780 ± 27	22
−5	22	90 ± 7*	2100 ± 53	22

*p < 0.01 vs. different order.

During baseline observations, in all animals (S and H groups) there were no changes in microvascular permeability and leukocyte adhesion; while SPV was 244.3 ± 11.0 nl/s and 240.2 ± 10.0 nl/s in S and H groups, respectively. Furthermore, all capillaries were perfused (Table 3).

**Table 3:** Changes in microvascular parameters of sham-operated (S) and hypoperfused (H) groups.

Group	Observation time	Diameter changes of order three arterioles (% reduction compared to baseline)	Microvascular Leakage (NGL)	Leukocyte adhesion (number of leukocyte/100 μm of venular length/30 s)	Single pial venule blood flow (nl/s)	Capillary perfusion (% reduction compared to baseline)
**S**	**Baseline**	0 ± 3	0.04 ± 0.02	1.0 ± 0.3	244.3 ± 11.0	0 ± 4
**H**	**Baseline**	0 ± 4	0.03 ± 0.01	1.0 ± 0.4	240.2 ± 10.0	0 ± 3
	**BCCAO**	16.5 ± 1.0*	0.24 ± 0.03*	5 ± 1*	70.5 ± 5.8*	55.5 ± 2.3*
	**Reperfusion**	12.0 ± 1.5*	0.48 ± 0.04*	8 ± 2*	159.1 ± 11.0*	50.8 ± 3.1*

*p < 0.01 vs. baseline and S group.

**Figure 3:**
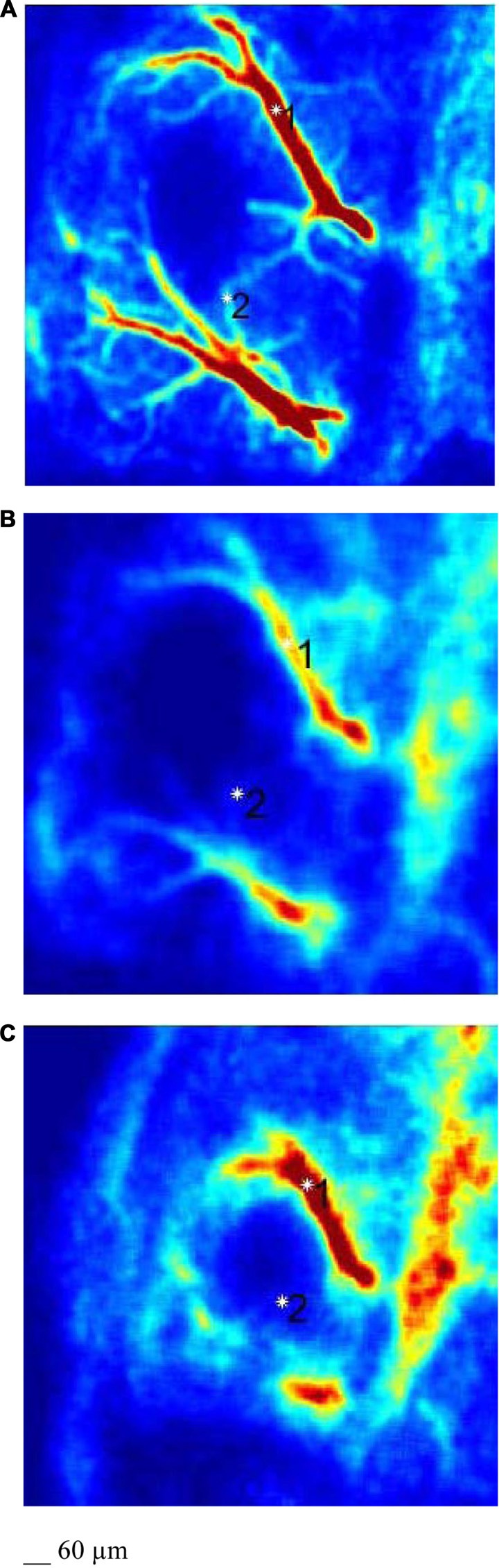
LSI images of a pial microvascular network under baseline conditions **(A)**, at the end of BCCAO **(B)** and at the end of reperfusion **(C)** in the same hypoperfused rat.

Rat pial microvascular images, derived from LSI, showed high (red) and low (blue) perfusion areas (Figure 3A). These images were compared with those obtained by fluorescence microscopy to identify each visualized vessel (Figures 2A,B): arterioles and venules of different orders were defined and the high flux areas corresponded to the largest vessels.

**Figure 4:**
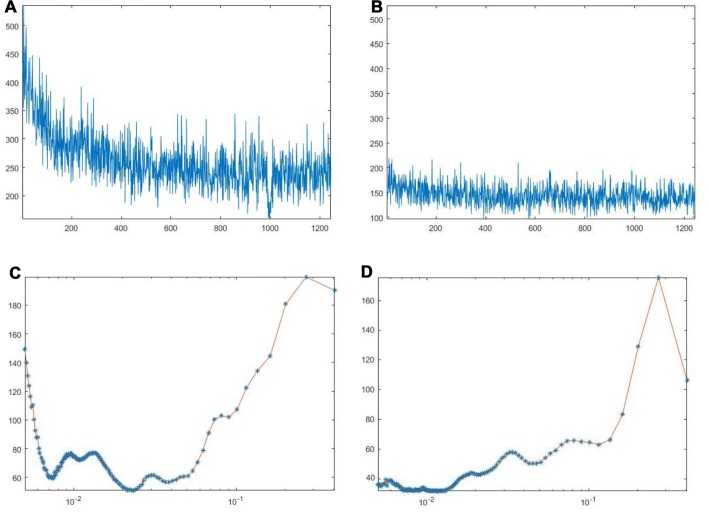
Arteriolar **(A)** and venular **(B)** blood flow oscillations and the main corresponding frequency components in arterioles **(C)** and venules **(D)** under baseline conditions. These data were obtained by Wavelet analysis of LSI image reported in Figure 3A.

Under baseline conditions, all vessels exhibited blood flow oscillations that were analyzed by spectral methods. A typical spectral analysis of arteriolar or venular single points in a laser speckle image is reported in Figure 4. The time-frequency plots (upper panels) were obtained using Wavelet transform. The lower panels display the spectra of the frequency components in the ranges 0.005–0.500 Hz. In particular, in 20 min recordings four frequency components were detected in the low frequency ranges: 0.005–0.0095 Hz, 0.0095–0.021 Hz, 0.021–0.052 Hz, 0.052–0.150 Hz. Another high frequency component was detected in the range: 0.150–0.500 Hz. During baseline observations, arterioles revealed significant higher total spectral density compared to venules (83 ± 5 PU2/Hz vs. 38 ± 4 PU^2^/Hz, respectively; *p* < 0.01; Figure 7). Moreover, the arteriolar frequency components in the ranges: 0.052–0.150 Hz and 0.150–0.500 Hz were predominant in the total power spectral density (PSD): 20 ± 2% and 74 ± 3% of total power spectrum, respectively. The remaining frequency components represented 6% ± 1% of total PSD (Figure 8A). In venules, the frequency component in the range 0.150–0.500 Hz presented the highest spectral density (>82.0 ± 1.5% of the total power), while the frequency component in the range 0.052–0.150 Hz showed lower spectral density when compared to the corresponding arteriolar frequency component (12.0 ± 0.5% vs. 20 ± 2% of total PSD; *p* < 0.05). Furthermore, the frequency components in the ranges 0.005–0.0095 Hz and 0.0095–0.021 Hz revealed negligible spectral density (1%–2% of the total power density; Figure 8B).

**Figure 5:**
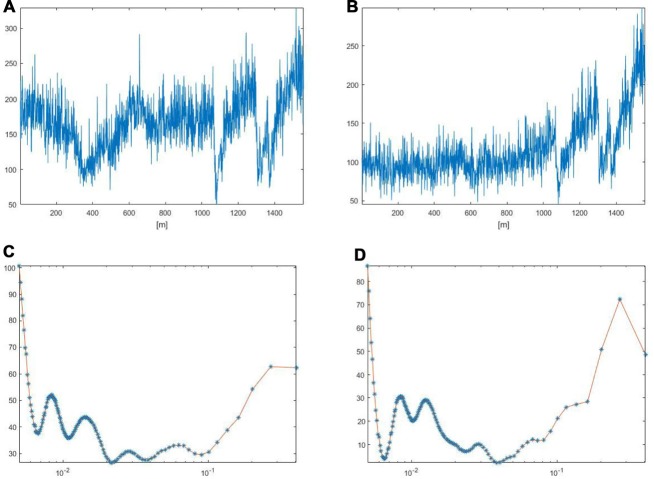
Arteriolar **(A)** and venular **(B)** blood flow oscillations and the main corresponding frequency components in arterioles **(C)** and venules **(D)** after 30 min BCCAO. These data were obtained by Wavelet analysis of LSI image reported in Figure 3B.

**Figure 6:**
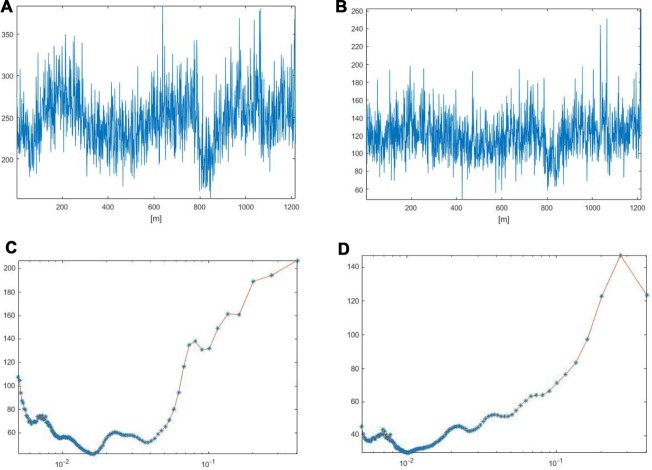
Arteriolar **(A)** and venular **(B)** blood flow oscillations and the main corresponding frequency components in arterioles **(C)** and venules **(D)** after 60 min reperfusion. These data were obtained by Wavelet analysis of LSI image reported in Figure 3C.

**Figure 7:**
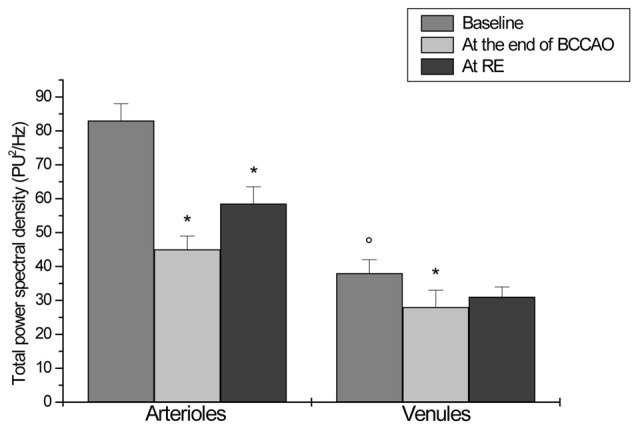
Total power spectral density (PSD), expressed as PU^2^/Hz, under baseline conditions, after 30 min BCCAO and at 60 min reperfusion in arterioles and venules of H group. *p < 0.01 vs. baseline, °*p* < 0.01 vs. arteriolar baseline value.

**Figure 8:**
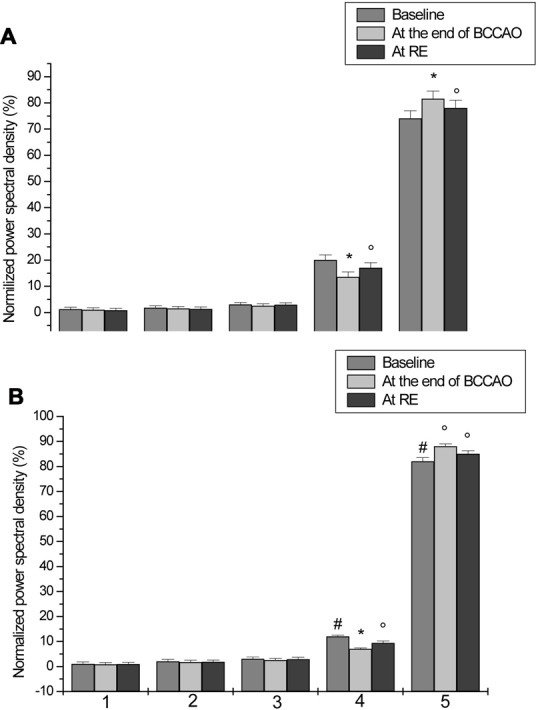
Normalized PSD, expressed as percent, in the ranges 0.005–0.0095 Hz (I), 0.0095–0.021 Hz (II), 0.021–0.052 Hz (III), 0.052–0.150 Hz (IV) and 0.150–0.500 Hz (V), under baseline conditions, after 30 min BCCAO and at 60 min reperfusion in arterioles **(A)** and venules **(B)** of H group. ^#^*p* < 0.05 venular vs. arteriolar baseline value, *p < 0.01 vs. baseline, °*p* < 0.05 vs. baseline.

### Bilateral Common Carotid Artery Occlusion (BCCAO)

During BCCAO, a dramatic reduction in arteriolar diameter, compared to baseline, was observed by fluorescence microscopy, indicating a severe condition of hypoperfusion. At the end of BCCAO, order 3 arteriole diameter was reduced by 16.5 ± 1.0% of baseline (*p* < 0.01 vs. baseline and S group). Moreover, fluorescent spots were detected along venular walls, demonstrating an increased microvascular permeability: NGL were 0.24 ± 0.03 (*p* < 0.01 vs. baseline and S group; Figure 2C, Table 3).

LSI images showed a reduction in the red areas, confirming the decreased blood perfusion levels (Figure 3B). Arteriolar and venular vessel oscillations, indeed, showed a significant decrease in total PSD (45 ± 4 PU2/Hz and 28 ± 5 PU^2^/Hz in arterioles and venules, respectively; *p* < 0.01 vs. baseline and S group), with a reduction in the percent spectral density of the low frequency components, when compared to baseline (Figures 5, 7). In particular, the arteriolar frequency component in the range 0.052–0.150 Hz revealed a significant reduction in spectral density; indeed, percent PSD was 13.5 ± 0.8% of total power spectrum (*p* < 0.01 vs. baseline and S group). The frequency component, in the range 0.150–0.500 Hz, increased in percent spectral density, compared to baseline (81.5 ± 1.0% vs. 74 ± 3%; *p* < 0.01 vs. baseline and S group), while the spectral density of the frequency components in the ranges 0.005–0.0095 Hz and 0.0095–0.021 Hz did not significantly change compared to baseline (Figure 8A). In venules, the frequency component in the range 0.052–0.150 Hz showed a significant decrease in the spectral density, as observed also in arterioles during BCCAO (7.0 ± 0.4% vs. 12.0 ± 0.5% of the total power; *p* < 0.01 vs. baseline and S group), while there was an increase in the percent spectral density of frequency component in the range 0.150–0.500 Hz (88 ± 1% vs. 82 ± 1.5%; *p* < 0.05 vs. baseline and S group). There were no significant variations in the other frequency components, compared to baseline, even though the trend was toward a decrease, without significant variations in the other frequency components (Figure 8B).

### Reperfusion

After the beginning of reperfusion, blood flow was restored and arteriolar diameter progressively increased, even though arteriolar constriction persisted up to the end of reperfusion: order 3 arteriole diameter decreased by 12.0 ± 1.5% of baseline (*p* < 0.01 vs. baseline and S group). At 60 min reperfusion, leakage of fluorescent tracer was more marked when compared with BCCAO values (NGL: 0.48 ± 0.04; *p* < 0.01 vs. baseline and S group). Furthermore, leukocyte adhesion was significant in the venules (8 ± 2/100 μm v.l./30 s; *p* < 0.01 vs. baseline and S group) and blood flow, calculated in the SPVs, was 159.1 ± 11.0 nl/s (*p* < 0.01 vs. baseline and S group), reduced by 33.8 ± 5% of baseline. Finally, PCL decreased by 50.8 ± 3.1% of baseline (*p* < 0.01 vs. baseline and S group; Figure 2D, Table 3).

LSI measurements indicated persistent low perfusion levels in pial microcirculation, whereas there was an increase in total spectral density of microvascular oscillations compared to BCCAO values (Figure 3C). However, at the end of reperfusion the total power density did not completely regain the baseline values: 58.8 ± 5 PU^2^/Hz and 31 ± 3 PU^2^/Hz in arterioles and venules, respectively (*p* < 0.01 vs. baseline and S group; Figures 6, 7). The arteriolar low frequency components (in the range 0.005–150 Hz) increased in their percent spectral density; particularly the frequency component in the range 0.052–0.150 Hz, that was 17.0 ± 0.6% of total PSD, compared to BCCAO (13.5 ± 0.8%; *p* < 0.05) and baseline (20 ± 2%; *p* < 0.01). Conversely, the percent spectral density of the frequency component in the range 0.150–0.500 Hz decreased, compared to BCCAO values (78 ± 2% vs. 81.5 ± 1.0%; *p* < 0.05 vs. BCCAO), but did not regain the baseline value (74 ± 3%; *p* < 0.05 vs. baseline and S group; Figure 8A). The venular blood flow increased, with a recovery in the percent spectral density of the frequency component in the range 0.052–0.150 Hz (9.4 ± 0.8% vs. 7.0 ± 0.4%; *p* < 0.05 vs. BCCAO); the percent spectral density of the frequency component in the range 0.150–0.500 Hz decreased compared to BCCAO value, but it was higher compared to baseline (85.0 ± 1.3% vs. 82.0 ± 1.5%; *p* < 0.05 vs. baseline and S group). The other frequency components did not significantly differ at the end of reperfusion, compared to baseline (Figure 8B).

### 2,3,5-Triphenyltetrazolium Chloride (TTC) Staining

**Figure 9:**
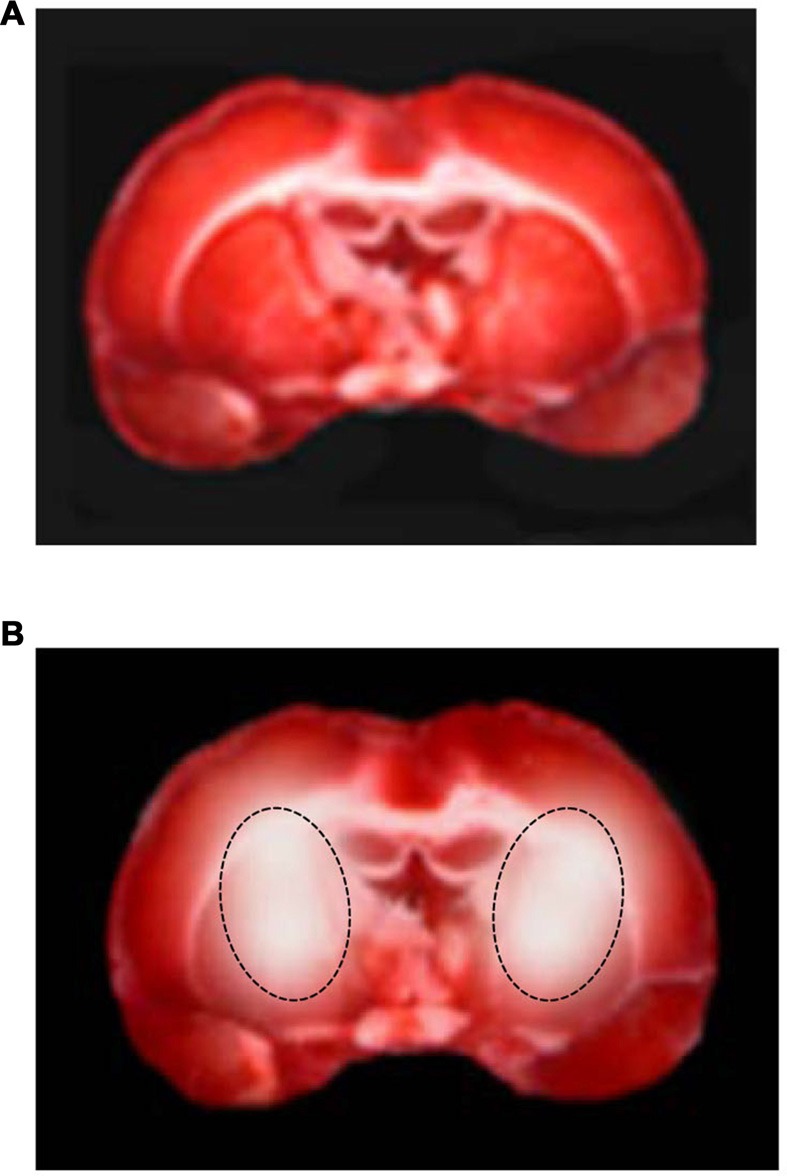
2,3,5-triphenyltetrazolium chloride (TTC) staining of coronal brain slices under baseline conditions **(A)** and at the end of reperfusion **(B)** in a hypoperfused rat. The lesion in the striatum is outlined by the dashed black line.

No necrotic area was detected in the animals belonging to S group, because their whole cerebral tissue was stained red. Conversely, the infarcted tissue was visible as the area of pallor in the brain slices of animals, submitted to 30 min BCCAO and 60 min reperfusion (H group). In particular, unstained tissue was mainly evident in the cortex and striatum, indicating neuronal loss (Figure 9).

## Discussion

The data of the present study indicate that 30 min BCCAO and subsequent 60 min reperfusion induced changes in rat pial microvascular blood flow. In particular, significant changes were detected in the spectral density of the frequency components, modulating the blood flow oscillations.

Fluorescence microscopy data show a decrease in arteriolar and venular blood flow with impairment of capillary perfusion, altered vessel wall integrity and increased leukocyte extravasation at the end of reperfusion. BCCAO model was introduced by Eklöf and Siesjö (1972, 1973) to cause cerebral transient hypoperfusion in experimental models. BCCAO and reperfusion, indeed, are able to determine significant microvascular alterations, such as arteriolar constriction with altered vasomotor tone, inflammatory processes and extracellular matrix degradation with consequent blood brain barrier disruption and cerebral edema, as previously reported (Lapi et al., 2012, 2015, 2016; Mastantuono et al., 2015, 2016b). Furthermore, these microvascular impairments are accompanied by infarct formation limited to the cortex and striatum, as detected by TTC staining.

In the present study, we used the innovative LSI technique combined with fluorescence microscopy to investigate the blood perfusion of cerebral tissue during hypoperfusion and reperfusion, obtaining high spatial and temporal resolution images. We compared LSI images with those acquired with fluorescence microscopy to identify arterioles and venules. LSI data confirmed the reduction in arteriolar and venular blood flow, because of the reduction in the red areas of arterioles and venules. However, it is worth noting that different changes in blood flow oscillations were detected during the different phases of the experimental protocol, analyzing blood flow in single vessels.

LSI has become widely used over the past decade; particularly, in animal stroke models, because it permits to quantify blood flow changes with high spatial and temporal resolution. Moreover, LSI technique combined with other optical techniques, such as hemoglobin oxygenation, has been utilized to simultaneously investigate blood flow and other cerebral hemodynamic parameters during stroke (Luo et al., 2009; Dunn, 2012). In addition, recently this technique has been used for real-time intraoperative monitoring of cerebral blood flow during neurosurgery in experimental models as well as in humans (Dunn et al., 2001; Dunn, 2012; Yuan et al., 2015). However, no data about temporal and spatial changes in cerebral blood flow oscillatory patterns have been reported.

Our data demonstrate that the spectral analysis carried out on single points of arterioles or venules, chosen in the laser speckle images, identified five frequency components in the ranges 0.005–0.500 Hz. The frequency components in blood flow oscillations have been related to specific physiological processes. In particular, in the oscillations of the blood flow in laser Doppler tracings derived from human skin, the several frequency components have been reported in the ranges 0.005–2 Hz. According to these observations, it has been suggested that these components are related to arterial wall cell functions. The frequency components in the ranges 0.005–0.0095 Hz and 0.0095–0.021 Hz were related to NO-independent and NO-dependent endothelial activity, respectively. Moreover, the components into ranges 0.021–0.052 Hz and 0.052–0.145 Hz were correlated to the neurogenic and the intrinsic myogenic activity of vascular smooth muscle cells, respectively. Finally, the ranges 0.145–0.6 Hz and 0.6–2.0 Hz have been associated to the respiration and the heart rates, respectively (Kvandal et al., 2003, 2006; Lapi et al., 2010). Our data indicate that oscillations in rat pial microcirculation presented quite the same ranges previously reported with the exception of the frequency component related to the heart rate.

Our data indicate that, under baseline conditions, total PSD was higher in arterioles when compared to venules, because of significant rhythmic changes in diameter observed in the arterioles (Ursino et al., 1998). It is well known, indeed, that these rhythmic changes in arteriolar lumen can modify blood flow, contributing to the characteristic oscillatory patterns observed in rat arterioles. It is worth noting that chloralose anesthesia did not completely abolish arteriolar diameter changes and consequent oscillations in blood flow (Colantuoni et al., 1984). Our results demonstrate that the major contribution under control conditions in arteriole blood flow oscillations was due to the frequency components in the ranges 0.052–0.150 Hz and 0.150–0.500 Hz, likely related to myogenic activity and respiration. Moreover, the respiration-related component was the highest in the spectral density also in the venules.

Our data did not include information on the frequency component that has been related to the heart rate (range: 0.500–4.0 Hz), because of the very low sampling rate (1 Hz). However, it is reasonable to suggest that information about the frequency component, related to heart rate, could be included in the spectral density of the frequency component in the range 0.150–0.500 Hz. Previously, both frequency components in the highest frequency ranges have been related to systemic mechanisms such as respiratory and heart rate.

However, the present date indicate that the frequency components related to endothelial and neurogenic activities presented, on the average, 6% of the total spectral density. On the other hand myogenic and systemic related components represented 94% of the total spectral density. These results do not support previous evaluation on the spectral density of blood flow or diameter oscillations in different experimental models. These discrepancies might be due to the calculations performed on the present data, where the sampling rate was reduced compared to laser Doppler data observed in previous studies, where the sampling was higher, thus facilitating the appropriate evaluation of oscillatory total spectral density.

We observed that the reduction in blood flow during ischemia and the recovery of tissue perfusion at the end of reperfusion were accompanied by corresponding decrease in total power density at the end of ischemia, with a significant decrease in the myogenic related component; in this condition respiration-related component was highly predominant. At reperfusion, the spectral density of the frequency components of arteriolar and venular blood flow oscillations increased, even though the values were lower than those observed under control conditions both in arterioles and venules. It is interesting to note that the percent increase in myogenic activity at the end of reperfusion was the highest. These results suggest that ischemia was accompanied by a decrease in myogenic activity, characterized by marked reduction in vessel diameter and oscillations. The recovery of blood flow was accompanied by the increase in vessel diameter and oscillations, as identified by fluorescent microscopy technique and spectral analysis carried out in single points of arterioles and venules.

Therefore, integrating fluorescent microscopy and LSI technique, these data demonstrate that hypoperfusion caused decrease in vessel diameter and corresponding reduction in blood flow oscillations, due to the decrease in vascular smooth muscle cell activity. To confirm these functional changes, we tried to identify a serum marker of vascular smooth muscle cell damage. However, further studies are required, because preliminary data did not present statistically significant differences. Reperfusion was accompanied by increase in vessel diameter and a rise in blood flow oscillations, related notably to the myogenic activity. Therefore, it is possible to suggest that redistribution of blood flow in brain circulation is related to arteriolar muscle cell activity.

## Author Contributions

TM, AC and DL: conceived and designed the experiments. TM, NS, LB, AG, AC and DL: performed the experiments and the animal treatments. TM, NS, LB, MDM, MCh, GN, EM, MCe, LI, GDA, AG, AC and DL: analyzed the data. TM, AC and DL: wrote the article.

## Conflict of Interest Statement

The authors declare that the research was conducted in the absence of any commercial or financial relationships that could be construed as a potential conflict of interest.
